# Supplementary material for: Actions Speak Louder Than Words: Health Behaviours and the Literacy of Future Healthcare Professionals
Source: Healthcare (Basel). 2022 Sep 8;10(9):1723. doi: 10.3390/healthcare10091723 (PMC9498724; doi:10.3390/healthcare10091723)
Supplement: Supplementary file 1 [file healthcare-10-01723-s001.zip › healthcare-1856643-supplementary.pdf]

Actions speak louder than words: Health behaviours and literacy of future healthcare professionals

Supplementary Material

Table S1. Predictors for HL indices – multivariate multinomial logistic regression

Outcome category	Explanatory variable	OR	95% CI for OR		p value
			Lower limit	Upper limit	
**Model for HL8 statement 1: *"I report any unusual signs and symptoms to my doctor or other healthcare professional"*** *p for the model=0.010* *Nagelkerke R^2=0.106, Cox-Snell R^2=0.097*					
„difficult"	Sex-male	7.91	0.98	63.9	0.052
	Sex-female *Reference category*	--	--	--	--
	Residence – 10.000-100.000 inhabitants	0.34	0.09	1.25	0.104
	Residence – above 100.000 inhabitants	1.33	0.32	5.53	0.691
	Residence – below 10.000 inhabitants *Reference category*	--	--	--	--
"easy"	Sex-male	2.65	0.31	22.20	0.370
	Sex-female *Reference category*	--	--	--	--
	Residence – 10.000- 100.000 inhabitants	0.60	0.17	2.14	0.428
	Residence – above 100.000 inhabitants	2.05	0.49	8.52	0.324
	Residence – below 10.000 inhabitants *Reference category*	--	--	--	--
"very easy"	Sex-male	3.05	0.36	26.1	0.309
	Sex-female *Reference category*	--	--	--	--
	Residence – 10.000- 100.000 inhabitants	0.65	0.16	2.59	0.536
	Residence – above 100.000 inhabitants	3.85	0.87	16.96	0.075
	Residence – below 10.000 inhabitants *Reference category*	--	--	--	--
**Model for HL8 statement 5*: "I ask healthcare professionals for information on how to take care of myself properly"*** *p for the model=0.0.009*					

Nagelkerke R^2=0.088, Cox-Snell R^2=0.0814					
"difficult"	Residence – 10.000- 100.000 inhabitants	0.78	0.33	1.87	0.579
	Residence – above 100.000 inhabitants	2.78	1.31	5.89	*0.008*
	Residence – below 10.000 inhabitants<br>*Reference category*	--	--	--	--
"easy"	Residence – 10.000- 100.000 inhabitants	1.33	0.53	3.34	0.534
	Residence – above 100.000 inhabitants	2.46	1.05	5.74	*0.037*
	Residence – below 10.000 inhabitants<br>*Reference category*	--	--	--	--
"very easy"	Residence – 10.000- 100.000 inhabitants	10.0	1.15	86.77	*0.036*
	Residence – above 100.000 inhabitants	9.5	1.10	82.45	*0.041*
	Residence – below 10.000 inhabitants<br>*Reference category*	--	--	--	--
**Model for HL8 statement 6: *"When I buy food, I read nutrition label"***<br>*p for the model= 6.683e-06*<br>*Nagelkerke R^2=0.269, Cox-Snell R^2=0.252*					
"difficult"	Sex-male	0.32	0.12	0.90	*0.031*
	Sex-female<br>*Reference category*	--	--	--	--
	Residence – 10.000- 100.000 inhabitants	2.51	0.90	6.98	0.078
	Residence – above 100.000 inhabitants	2.04	0.75	5.51	0.160
	Residence – below 10.000 inhabitants<br>*Reference category*	--	--	--	--
	Major – Radiography	2.02e-06	5.90e-07	6.91e-06	*<0.0001*
	Major – Physiotherapy	1.44e-06	3.70e-07	5.58e-06	*<0.0001*
	Major – Medicine	1.56e-06	4.51e-07	5.37e-06	*<0.0001*
	Major – Nursing	5.86e-07	2.14e-07	1.60e-06	*<0.0001*
	Major – Public Health	3.17e-06	9.11e-07	1.10e-05	*<0.0001*
	Major – Dietetics<br>*Reference category*	--	--	--	--
"easy"	Sex-male	0.28	0.11	0.76	*0.012*
	Sex-female<br>*Reference category*	--	--	--	--

	Residence – 10.000- 100.000 inhabitants	0.91	0.33	2.56	0.866
	Residence – above 100.000 inhabitants	1.93	0.77	4.83	0.157
	Residence – below 10.000 inhabitants *Reference category*	--	--	--	--
	Major – Radiography	1.1e-06	3.44e-07	3.54e-6	*<0.0001*
	Major – Physiotherapy	1.62e-06	4.97e-07	5.26e-06	*<0.0001*
	Major – Medicine	6.89e-07	2.05e-07	2.3e-06	*<0.0001*
	Major – Nursing	4.84e-07	1.98e-07	1.18e-06	*<0.0001*
	Major – Public Health	1.99e-06	6.12e-07	6.47e-06	*<0.0001*
	Major – Dietetics *Reference category*	--	--	--	--
"very easy"	Sex-male	0.44	1.46e-01	1.33e+00	0.145
	Sex-female *Reference category*	--	--	--	--
	Residence – 10.000- 100.000 inhabitants	3.65	1.01	13.18	*0.048*
	Residence – above 100.000 inhabitants	4.21	1.27	14.01	*0.019*
	Residence – below 10.000 inhabitants *Reference category*	--	--	--	--
	Major – Radiography	1.12e-07	2.18e-08	5.76e-07	*<0.0001*
	Major – Physiotherapy	3.67e-07	1.01e-07	1.33e-06	*<0.0001*
	Major – Medicine	4.87e-07	1.56e-07	1.52e-06	*<0.0001*
	Major – Nursing	1.12e-07	4.26e-08	2.93e-07	*<0.0001*
	Major – Public Health	1.63e-07	3.65e-08	7.33e-07	*<0.0001*
	Major – Dietetics *Reference category*	--	--	--	--
**Model for HL8 statement 7: *"I participate in educational programmes on how to take care of my health"*** *p for the model*= 0.107 *Nagelkerke R^2=0.138, Cox-Snell R^2=0.124*					
"difficult"	Major – Radiography	0.16	0.03	0.73	*0.018*
	Major – Physiotherapy	0.31	0.07	1.33	0.114
	Major – Medicine	0.38	0.09	1.69	0.206
	Major – Nursing	0.23	0.06	0.91	*0.036*
	Major – Public Health	0.23	0.05	1.00	*0.049*
	Major – Dietetics *Reference category*	--	--	--	--

"easy"	Major – Radiography	0.14	0.03	0.72	*0.018*
	Major – Physiotherapy	0.03	0.002	0.29	*0.003*
	Major – Medicine	0.15	0.03	0.81	*0.028*
	Major – Nursing	0.12	0.02	0.43	*0.002*
	Major – Public Health	1.44e-07	0.04	0.87	*0.033*
	Major – Dietetics<br>*Reference category*	--	--	--	--
"very easy"	Major – Radiography	9.82e-09	9.82e-09	9.83e-09	*<0.0001*
	Major – Physiotherapy	1.92e-08	1.92e-08	1.92e-08	*<0.0001*
	Major – Medicine	0.09	6.75e-03	1.22	0.070
	Major – Nursing	0.12	0.017	0.79	*0.028*
	Major – Public Health	1.45e-07	*	*	0.981
	Major – Dietetics<br>*Reference category*	--	--	--	--
**Model for HL8 statement 8: "*When needed, I use counselling or therapy services*"**<br>*p for the model= 0.035*<br>*Nagelkerke R^2=0.130, Cox-Snell R^2=0.120*					
"difficult"	Major – Radiography	0.44	0.11	1.8	0.252
	Major – Physiotherapy	0.54	0.13	2.2	0.394
	Major – Medicine	0.70	0.16	2.98	0.639
	Major – Nursing	0.34	0.09	1.23	0.100
	Major – Public Health	0.76	0.18	3.11	
	Major – Dietetics<br>*Reference category*	--	--	--	--
"easy"	Major – Radiography	0.24	0.05	1.13	0.07
	Major – Physiotherapy	0.04	0.003	0.43	*0.008*
	Major – Medicine	0.56	0.12	2.49	0.442
	Major – Nursing	0.23	0.06	0.88	*0.032*
	Major – Public Health	0.39	0.09	1.80	0.229
	Major – Dietetics<br>*Reference category*	--	--	--	--
"very easy"	Major – Radiography	1.97e-07	*	*	0.981
	Major – Physiotherapy	0.27	0.04	1.79	0.176
	Major – Medicine	0.25	0.03	2.00	0.191
	Major – Nursing	0.59	0.13	2.7	0.499
	Major – Public Health	0.67	0.12	3.75	0.646
	Major – Dietetics	--	--	--	--

	Reference category				

* 95% CI estimated as (0.00; Inf) due to a very small number of cases

Table S2. Predictors for BMI in the cohort – multivariate linear regression

Independent variable	B	S.E. for B	95% CI for B		*p value*
			Lower limit	Upper limit	
Sex-male	2.52	0.49	1.55	3.5	*7.24e-07*
Sex-female<br>*Reference category*	--	--	--	--	--
Major – Radiography	1.77	0.78	0.21	3.3	*0.026*
Major – Physiotherapy	-0.41	0.8	-1.98	1.16	0.606
Major – Medicine	0.136	0.78	-1.41	1.68	0.862
Major – Nursing	1.70	0.65	0.42	2.99	*0.009*
Major – Public Health	1.76	0.73	0.32	3.20	*0.017*
Major – Dietetics<br>*Reference category*	--	--	--	--	--

p for the model=1.99e-06, R^2=0.123

Table S3. Predictors for HBI subscales – multivariate linear regression

Independent variable	B	S.E. for B	95% CI for B		*p value*
			Lower limit	Upper limit	
Model for HBI Total<br>*p for the model=0.029, R^2=0.03*					
Sex-male	-3.85	1.78	-7.35	-0.35	*0.031*
Sex-female<br>*Reference category*	--	--	--	--	--
Monthly income – 1000-2000 PLN	-4.55	2.15	-8.79	-0.31	*0.035*
Monthly income – above 2000 PLN	-2.65	2.19	-6.95	1.65	0.226
Monthly income – below 1000 PLN<br>*Reference category*	--	--	--	--	--
Model for HBI Proper Eating Habits<br>*p for the model= 5.22e-08, R^2=0.162*					
Sex-male	-0.43	0.11	-0.66	-0.21	*0.0002*
Sex-female<br>*Reference category*	--	--	--	--	--

Residence – 10.000- 100.000 inhabitants	0.21	0.12	-0.03	0.45	0.088
Residence – above 100.000 inhabitants	0.25	0.11	0.03	0.46	*0.023*
Residence – below 10.000 inhabitants<br>*Reference category*	--	--	--	--	--
Major – Radiography	-0.76	0.18	-1.11	-0.38	*6.29e-05*
Major – Physiotherapy	-0.61	0.18	-0.97	-0.25	*0.001*
Major – Medicine	-0.5	0.18	-0.86	-0.15	*0.006*
Major – Nursing	-0.71	0.16	-1.02	-0.41	*7.83e-06*
Major – Public Health	-0.81	0.17	-1.15	-0.48	*2.44e-06*
Major – Dietetics<br>*Reference category*	--	--	--	--	--
Model for HBI Healthy Practices<br>*p for the model= 0.018,* R^2*=0.019*					
Sex-male	-0.23	0.04	-0.42	-0.04	*0.018*
Sex-female<br>*Reference category*	--	--	--	--	--
